# National policy actions on dementia in the Americas and Asia-Pacific: Consensus and challenges

**DOI:** 10.26633/RPSP.2020.2

**Published:** 2020-01-22

**Authors:** Fei Sun, Emmanuel Chima, Tracy Wharton, Vijeth Iyengar

**Affiliations:** 1 School of Social Work Michigan State University East LansingMichigan United States of America School of Social Work, Michigan State University, East Lansing, Michigan, United States of America.; 2 School of Social Work University of Central Florida OrlandoFlorida United States School of Social Work, University of Central Florida, Orlando, Florida, United States.; 3 Administration on Aging/Administration for Community Living United States Department of Health and Human Services WashingtonDC United States Administration on Aging/Administration for Community Living, United States Department of Health and Human Services, Washington, DC, United States.

**Keywords:** Dementia, health policy, Alzheimer disease, Americas, Asia, Demencia, política de salud, enfermedad de Alzheimer, Américas, Asia, Demência, política de saúde, doença de Alzheimer, Américas, Asia

## Abstract

Alzheimer’s disease and related dementias (ADRD) affect over 50 million persons globally, and the number is expected to rise. In response, health ministries are developing and implementing policies and programs to systemically address the needs of individuals and families affected by ADRD. While national plans of action on ADRD are advancing among European Member States of World Health Organization (WHO), those in the Asia-Pacific and Americas are lagging behind. Since previous studies have largely ignored the Americas and Asia-Pacific—where approximately two-thirds of the global ADRD population resides—this study sought to identify (a) the socioeconomic factors associated with the likelihood of having a national dementia policy, and (b) to examine common and differing features among the national plans in these regions. Employing the dementia policy guidelines of WHO and the Pan American Health Organization as an extraction guide for data collection and analysis, the national dementia plans and available socioeconomic data of 10 Member States were analyzed with comparative and qualitative analyses. Findings suggested at least a 14-fold increase in the likelihood of having a national dementia plan if a Member State had one of the following: a universal health care system, more than 14% of the population 65 years of age or older, or high-income. All the Member States in the study identified dementia as a public health priority, but priorities differed. Inconsistencies included development of information systems, training for health care professionals, and long-term care systems.

## RESUMEN

La enfermedad de Alzheimer y otras demencias relacionadas afectan a más de 50 millones de personas a nivel mundial y se espera que esta cifra aumente. En respuesta, los ministerios de salud están elaborando y ejecutando políticas y programas para abordar de manera sistemática las necesidades de las personas y familias afectadas. Si bien los planes nacionales de acción sobre la enfermedad de Alzheimer y otras demencias progresan en los Estados Miembros europeos de la Organización Mundial de la Salud (OMS), los de las regiones de Asia y el Pacífico y las Américas están a la zaga. Puesto que en anteriores estudios se ha pasado por alto en gran parte a las regiones de las Américas y Asia y el Pacífico, donde residen aproximadamente dos terceras partes de la población mundial con estas enfermedades, en este estudio se ha tratado de: *a*) determinar los factores socioeconómicos relacionados con la probabilidad de contar con una política nacional en materia de demencia, y *b*) examinar las características comunes y distintas de los planes nacionales en esas regiones. Se examinaron los planes nacionales sobre demencia y los datos socioeconómicos disponibles de diez Estados Miembros mediante análisis comparativos y cualitativos en los que se emplearon las directrices para la formulación de políticas en materia de demencia de la OMS y la Organización Panamericana de la Salud a modo de guía de extracción para la recopilación y el análisis de datos. Los resultados indicaron que había un aumento de las probabilidades de al menos 14 veces de disponer de un plan nacional en materia de demencia siempre que el estado miembro contase con al menos uno de los siguientes elementos: un sistema de atención de salud universal, más de 14% de su población en la edad de 65 años o más, o ingresos elevados. Todos los Estados Miembros del estudio establecían la demencia como una cuestión prioritaria de salud pública, si bien las prioridades diferían. Entre las diferencias se encontraban el desarrollo de los sistemas de información, la capacitación de los profesionales de la salud y los sistemas de cuidados a largo plazo.

## RESUMO

A doença de Alzheimer e demências relacionadas (DADR) afetam mais de 50 milhões de pessoas em todo o mundo, e este número deverá aumentar no futuro. Em resposta, os ministérios da saúde estão desenvolvendo e implementando políticas e programas para atender sistematicamente às necessidades das pessoas e famílias afetadas pela DADR. Embora os planos de ação nacionais para a DADR estejam avançando entre os Estados Membros europeus da Organização Mundial da Saúde (OMS), os das regiões das Américas e Ásia-Pacífico estão ficando para trás. Estudos anteriores ignoraram amplamente as regiões das Américas e Ásia-Pacífico, onde encontram-se aproximadamente dois terços da população mundial com DADR; por isso, este estudo procurou (a) identificar os fatores socioeconômicos associados à probabilidade de que um país conte com uma política nacional para demência e (b) examinar as características comuns e diferentes dos planos nacionais existentes nessas regiões. Empregando as diretrizes para políticas sobre demência da OMS e da Organização Pan-Americana da Saúde como um guia para a coleta e análise de dados, examinamos os planos nacionais para demência e os dados socioeconômicos disponíveis em 10 Estados Membros, realizando análises comparativas e qualitativas. Os resultados sugeriram um aumento de pelo menos 14 vezes na probabilidade de que um Estado Membro conte com um plano nacional para demência quando esse Estado Membro apresenta um dos seguintes fatores: um sistema de atenção universal à saúde, mais de 14% da população com 65 anos de idade ou mais, ou alta renda. Todos os Estados Membros incluídos no estudo identificaram a demência como uma prioridade de saúde pública, mas com prioridades distintas. As diferenças incluíram o desenvolvimento de sistemas de informação, a formação oferecida aos profissionais da saúde e os sistemas de atenção à saúde de longa duração.

Approximately 50 million individuals currently live with Alzheimer disease or related dementias (ADRD) worldwide (1). In responding to the growing needs of people living with ADRD, countries are at various stages of developing and implementing national dementia plans. Existing national governmental plans, sub-national plans (e.g., provincial or state governmental plans), and non-governmental national strategies reflect a collective commitment to strategically addressing the impact of ADRD on society. These strategies touch on various aspects of dementia diagnosis, treatment, and care; education and support for family caregivers; training for health and social care professionals; and accommodations in physical and social environments (2).

The World Health Organization (WHO) has launched an 8-year plan, the “Global Action Plan on the Public Health Response to Dementia, 2017 – 2025,” (3) to provide guidance to Member States that are developing national plans and to offer an evaluation framework for those with plans in place. In December 2018, there were 27 national dementia plans available across five of the six WHO Regions: 16 plans in the European Region (EURO); 5 in the Region of the Americas (AMRO); 4 in the Western Pacific Region (WPRO); 1 in the Eastern Mediterranean Region (EMRO); and 1 in the South-East Asia Region (SEARO) (4, 5). To the best of our knowledge, the Africa Region (AFRO) does not have any plans in place yet.

Since EURO has approximately 60% of the national dementia plans, available reviews have focused primarily on progress in this region (6, 7). Highlighting this observation, a 2013 report by the Blue Cross and Blue Shield Association (Chicago, Illinois, United States) and Alzheimer’s Disease International (ADI; London, United Kingdom) examined best practices in seven national dementia plans available at the time, including only three plans outside of Europe (6). This paper, however, examines a broader scope of currently available plans by focusing on Member States in AMRO and the Asia-Pacific regions, SEARO and WPRO.

Approximately 32 million persons with dementia live in the Americas and the Asia-Pacific (8, 9)—accounting for two-thirds of world’s dementia population—but only 10 Member States (4) in these regions have national dementia plans: Australia, New Zealand, Japan, Republic of Korea, Indonesia, Chile, Costa Rica, Cuba, Mexico, and the United States. This constitutes < 10% of the countries in these regions.

The objective of this study was to examine the existing national dementia plans in the Americas and Asia-Pacific regions to: (a) identify the socioeconomic factors associated with having a plan and (b) to assess the common and differing features of existing plans through comparative and qualitative analyses.

## METHODS AND MATERIALS

Data were drawn from publicly available resources and datasets, including ADI reports, published articles, and the World Bank. In all, 71 sovereign countries (10) of 96 Member States and territories in the Americas (AMRO) and Asia-Pacific regions (both SEARO and WPRO) were selected for this study (5).

To identify the socioeconomic factors associated with having a national dementia plan, it was postulated that a Member State’s characteristics in the political, economic, demographic, and health care domains would affect plan adoption. Data was collected on each country’s political system, i.e., democracy vs. non-democracy (11); economic classification, based on gross national income per-capita (12); demographics, e.g., percent population of older adults (13); and health care coverage, i.e., a universal health care system or not (14). Logistic regression analyses were conducted to identify associations with the likelihood of having a national dementia policy.

To assess the common and differing features of existing plans, a framework was adapted from the WHO Global Action Plan (3) and the PAHO Strategy and Plan of Action on Dementias in Older Persons (15) to guide the qualitative review. The WHO Plan outlines seven action areas: public health priority, awareness and friendliness, risk reduction, health services, caregiver support, information systems, and research (3). The PAHO Plan highlights 10 similar action areas: stigma reduction, health services, caregiver supports, research, policy development, supportive services, community supports, service provider evaluation, health professional training, and national action and policy indicators (15). Anticipating potential overlaps in the two plan’s action areas (e.g., raise awareness, improve health services, boost research and surveillance), the analyses specifically examined each national dementia plan against each indicator described by WHO and PAHO.

## RESULTS

### Factors associated with likelihood of having a national dementia plan

Table 1 presents the relationship between each of the four socioeconomic dimensions and the likelihood of a national dementia plan. Analysis of the political domain showed no relationship to the likelihood of having a national dementia plan; however, economic development revealed a statistically significant difference between high-income Member States and those of low or middle income. High income Members States were about 14 times (Odds Ratio = 14.4) more likely to have a national dementia plan. Additionally, if ≥ 14% of the population was 65 years of age or older, the Member State was more likely to have a national dementia plan; if that population was ≤ 10%, it was less likely to have a plan. Lastly, the presence— more so than the absence—of a universal health care system was associated with a higher likelihood of having a national dementia plan.

### Commonalities across national dementia plans

Ten of the existing national dementia plans in the Americas and the Asia-Pacific regions were analyzed. These plans set goals related to providing support and services for persons with dementia and their families. Provision of health services, community-based care, support for caregivers, increase in public dementia literacy, and decreased stigma were common goals highlighted by these plans (Table 2).

All 10 plans included increasing awareness and reducing the stigma of dementia as target goals. Indicators 1, 2, and 5 from the PAHO Plan (15)—calling for specific guidelines, protocols, and policies to address dementia care and stigma reduction— appeared in all 10 national plans. Nine plans (except for New Zealand), identified dementia research as a goal. Seven national plans (Chile, Costa Rica, Cuba, Indonesia, Mexico, Republic of Korea, and United States) mentioned an information system for tracking and sharing dementia-related data, knowledge, and progress within and/or among countries.

**TABLE 1.** Characteristics associated with the likelihood of having a National Plan on Dementia in Member States of the World Health Organization in the Americas and Asia-Pacific regions, 2019

Dimensions	Has a National Plan on dementia (*n* = 71)	
	Odds Ratio	Confidence Intervals
Political—Democracy	0.70	0.08 – 7.70
Economic level^1^		
Upper-middle income	2.67	0.26 – 27.38
High income	14.40^2^	1.53 – 135.51
Elder population percentage^3^		
≥ 65 years is 10% – 13.99%	4.64	0.59 – 36.58
≥ 65 years is ≥ 14%	21.25^4^	3.36 – 134.49
Health care—universal health care	19.67^4^	2.96 – 130.68

1 Reference group is low- and middle-income Member States.

2 *P* < 0.05

3 Reference group is Member States with population ≥ 65 years at 10% or less of the total population.

4 *P* < 0.01

***Source***: Prepared by the authors from the study results.

**TABLE 2.** Comparison of Pan American Health Organization (PAHO) Indicators and World Health Organization (WHO) Action Areas by Member States of the Americas (AMRO), Southeast Asia (SEARO), and Western Pacific (WPRO), 2019

PAHO Indicators and WHO Action Areas	Member States of the WPRO and SEARO Regions					Member States of the AMRO Region				
	Australia	Indonesia	Japan	Republic of Korea	New Zealand	Chile	Costa Rica	Cuba	Mexico	United States
PAHO-1 Specific intervention to reduce stigma/stereotypes & improve understanding	+	+	+	+	+	+	+	+	+	+
PAHO-2 Guidelines/protocols for care of PWD^1^	+	+	+	+	+	+	+	+	+	+
PAHO-3 Guidelines/protocols for health promotion & risk prevention and reduction with life course approach	?	+	+	+	?	+	+	+	+	+
PAHO-4 Evidence-based community intervention to help maintain functional capacity/independently	+	?	+	+	+	+	+	+	+	+
PAHO-5 Quality, integrated, community-based networks for the care of dependent persons	+	+	+	+	+	+	+	+	+	+
PAHO-6 Care and training programs for carers	+	+	+	+	+	+	+	+	+	+
PAHO-7 Continuous evaluation system for providers of long-term care	?	–	–	+	?	+	+	–	–	+
PAHO-8 Basic competencies in undergraduate and graduate education and continued training for social and health service personnel	–	+	–	+	?	+	+	+	+	+
PAHO-9 Indicators of dementias, disability, dependence, and long-term care	–	+	+	+	+	+	–	+	?	+
PAHO-10 Conducted national research studies on dementia	+	+	+	+	–	+	+	+	?	+
WHO-1 Dementia as a public health priority	+	+	+	–	+	+	+	+	+	+
WHO-2 Dementia awareness and friendliness	+	+	+	+	+	+	+	+	+	+
WHO-3 Dementia risk reduction	+	+	+	+	+	+	–	+	+	+
WHO-4 Dementia diagnosis, treatment, care, and support	+	+	+	+	+	+	+	+	+	+
WHO-5 Support for dementia carers^2^	+	+	+	+	+	+	+	+	+	+
WHO-6 Information systems for dementia	-	+	?	+	?	+	+	+	+	+
WHO-7 Dementia research and innovation	+	+	+	+	-	+	+	+	+	+

1 PWD refers to persons living with dementia.

2 Carer is an unpaid caregiver.

**Key**

+ indicates this Member State’s plan addresses this indicator or action area.

– indicates no mention of this indicator or action area.

? indicates partial or implicit mention of this indicator or action area.

***Source***: Prepared by the authors from the study results.

Nine national plans (except Republic of Korea) specifically highlighted dementia as a public health priority; of these, eight (except Costa Rica) included risk reduction, a common public health approach for disease prevention. Furthermore, all 10 plans address goals regarding supportive services for family caregivers, e.g., integrated community support networks and training for non-professional caregivers.

Given the projected economic impact that family caregivers have on providing care, supporting them is paramount (9, 16, 17). According to data from 2015, the per-person cost of caring for individuals with dementia ranged from US$ 872 in South Asia to US$ 3 375 in the Andean area of South America to US$ 56 218 in high-income countries of North America. Across regions, the informal care cost contributed by family members is about twice the combined health and social care cost spent on individuals with dementia through formal mechanisms (9). One systematic review found that in 2017 the annual informal care cost for dementia in Asia-Pacific and North America was US$ 109.9 billion and US$ 268.9 billion, respectively (17).

### Differences across national dementia plans

National policies that systematically address dementia, from prevention, diagnosis, and treatment, to care and supportive services are ideal; but Member States differed on priorities, goals, and strategies. Only Chile and the United States had national plans that covered all the domains. One obvious difference across plans was related to long-term care. Seven national plans referred to establishing indicators for long-term care quality, but only four specified a mechanism for evaluating long-term care providers.

Differences in other areas were more subtle. For example, eight Member States stipulated developing health promotion and risk reduction guidelines and protocols, with a life-course approach as a target goal. Notably, however, Australia and New Zealand’s plans did not contain this explicit target. Australia had a priority area on awareness raising and risk reduction, but without a life-course perspective. Given the shortage of professionals specialized in dementia care (18), workforce preparation and training were emphasized in most national plans; but only seven explicitly mentioned training at the university level or beyond.

## DISCUSSION

A national response and a government plan for addressing the growing challenges of dementia are imperative to setting a standard for all stakeholders. National plans provide a template for unifying efforts and driving progress. Additionally, plans are indicative of heightened governmental awareness and sustained commitment to issues despite changes in political administrations. Some WHO Regions have more fully developed, cohesive strategies than others. It is worth considering how the presence of a national plan may prompt real action, and whether actions are as disparate as they seem to be in the available documents.

Distinct differences in economic development, demographics, and health care systems can influence national policies, dementia efforts, and priority setting among countries. This is most apparent in that nearly all of the existing national dementia plans (4) are in high-income Member States (12). Furthermore, countries with a higher percentage of the population 65 years of age or older and those with a universal health care system are more likely to have a national dementia plan. Notably, however, more than one-half of the global population currently affected by dementia lives in low- and middle-income countries (1); by 2050, it could be 75% (9).

As demographics continue to shift, there will be a pressing need for policymakers in all low- and middle-income countries to address the issue from a national policy perspective. We caution, however, that cultural differences and geographic nuances need to be carefully considered. Previous research has highlighted this point, and called for more accurate dementia prevalence data (7). We recommend that each Member State develop its dementia policy in relation to its demographic, economic, cultural, and health care contexts. To get started, countries might develop a plan for areas with highly concentrated needs, addressing unique cultural dynamics or geographic restraints; or incorporate dementia care components into existing public health policies that can be impactful and lead to a national plan.

For the 10 Member States in this study that have national dementia plans, we postulate that different levels of dementia awareness and literacy, along with cultural perceptions of dementia, may explain variations in their approach to the WHO and PAHO guidelines. Again, interpreting findings must be done with caution. This analysis only begins to understand the policy actions taken to address dementia; it does not cover what implementation looks like “on the ground” in each country, nor what degree of real impact the plan actions have made. For example, although the national plans of the Chile and United States touch on all the WHO and PAHO recommendations and frameworks, how well or how broadly they were implemented was not examined by this study. Moreover, in the United States, policy implementation occurs at the state level, which means strategies and priorities could vary greatly across the country.

On the other hand, both the Republic of Korea and Japan did not address all aspects in the frameworks (see Table 2), which does not necessarily mean that their national policies had less impact. Indeed, the Republic of Korea had its first national dementia plan in 2008, four years before the United States, and its dementia management system has multi-level coverage along with integrated long-term care (19). Furthermore, national policy in Japan focuses on coordinating the continuum of care for dementia patients and on training dementia specialists (20). The plans of these two Member States are consistent with the cultural values of community-based care and the integration of family and social networks of support.

Another approach for advancing progress globally involves development of mechanisms for information sharing related to data, best practices in various care provision contexts, and policy approaches. The proliferation of dementia research, coupled with the projections of persons affected by dementia, make a systematic means of information-sharing imperative among the Member States. A system is needed that goes beyond the relatively slow nature of peer-reviewed publication processes. If more Member States invested in information systems, fragmented dissemination of information and uneven progress could be avoided.

The PAHO and WHO guidelines are a tool to identify commonly shared goals. They provide a framework within which each Member State can reflect on its strengths and uniqueness in their context, and thus can be used for assessing variance. Although all 10 Member States had reached a consensus on making dementia a national priority, the cultural perspective, social welfare values, and health care system options undoubtedly influenced the strategies and priorities within those plans. While some plans tended to focus on care and treatment of persons with dementia, others focused on the protection of human rights, inclusion, and equality, striving to offer persons with dementia broad engagement and opportunities, such as employment support.

Given that our analyses used publicly available resources and datasets, we may not have captured the most recent policy efforts. In fact, when examining their most recent status (4), 17 Member States in the AMRO, SEARO, and WPRO Regions have national plans under development. But even more notable is that 61 of these Member States (86%) do not have a national dementia plan in place or in development. Given that the first goal in the WHO Global Action Plan is that 75% of Member States will have a national response to dementia by 2025 (3), this dearth of plans underscores an urgency for more progress.

## Conclusion

A country’s economic development status, proportion of aging population, and availability of national health coverage are related to the likelihood of having a national dementia policy. While the Member States in this study had all identified dementia as a public health priority, requiring increased awareness and reduction of stigma, the policy priorities differed across nations in areas of training of health and social care professionals, development of information sharing systems, and reforming long-term care systems. We recommend that all national policies on dementia include better coordination of information systems, education of health professionals, and implementation of an effective and sustainable long-term care system.

## Author contributions.

FS and VI conceived the original idea. EC collected data with contributions from VI. TW analyzed data and drafted the related results and discussion. FS, EC, and VI wrote the paper. All authors reviewed and approved the final version.

## Acknowledgements.

The authors appreciate the critical feedback offered by Katrin Seeher (Department of Mental Health and Substance Abuse, World Health Organization).

## Disclaimer.

Authors hold sole responsibility for the views expressed in the manuscript, which may not necessarily reflect the opinion or policy of the *RPSP/PAJPH*, PAHO, the U.S. Administration for Community Living/Administration on Aging, and/or the U.S. Department of Health and Human Services.

## References

[1] 1. Alzheimer’s Disease International. Dementia statistics, 2018. Available from: https://www.alz.co.uk/research/statistics Accessed 12 November 2019.

[2] 2. Wortmann M. Importance of national plans for Alzheimer’s disease and dementia. Alzheimers Res Ther. 2013;5(5):40. 10.1186/alzrt205 PMC3978679 24007939

[3] 3. World Health Organization. Global Action Plan on the Public Health Response to Dementia, 2017–2025. Available from: https://apps.who.int/iris/bitstream/handle/10665/259615/9789241513487-eng.pdf Accessed 15 November 2019.

[4] 4. Alzheimer’s Disease International. Dementia plans. Available from: https://protect2.fireeye.com/url?k=5476a2ce-0822bbb2-547693f1-0cc47adc5fa2-23b0796b4aa3f213&u=https://www.alz.co.uk/dementia-plans Accessed 15 November 2019.

[5] 5. World Health Organization. WHO Regional Offices. 2019. Available from: https://www.who.int/about/who-we-are/regional-offices Accessed 15 November 2019.

[6] 6. Pot AM, Petrea I. BUPA/ADI report. Improving dementia care worldwide: Ideas and advice on developing and implementing a National Dementia Plan. London: Bupa/ADI October 2013. Available from: https://www.alz.co.uk/sites/default/files/pdfs/global-dementia-plan-report-ENGLISH.pdf Accessed 15 November 2019.

[7] 7. Wu YT, Fratiglioni L, Matthews FE, Lobo A, Breteler MM, Skoog I, Brayne C. Dementia in western Europe: epidemiological evidence and implications for policy making. Lancet Neurol. 2016;15(1):116-24. 10.1016/S1474-4422(15)00092-7 26300044

[8] 8. Alzheimer’s Disease International. Dementia in the Asia Pacific Region, 2014. Available from: https://www.alz.co.uk/dementia-in-the-asia-pacific Accessed 15 November 2019.

[9] 9. Alzheimer’s Disease International. World Alzheimer Report 2015: The Global Impact of Dementia. Available from: https://www.alz.co.uk/research/WorldAlzheimerReport2015.pdf Accessed 15 November 2019.

[10] 10. Bureau of Intelligence and Research, United States Department of State. Independent States in the World, 2019. Available from: https://www.state.gov/independent-states-in-the-world Accessed 15 November 2019.

[11] 11. Central Intelligence Agency. The World Factbook. Available from: https://www.cia.gov/library/publications/the-world-factbook/fields/299.html Accessed 15 November 2019.

[12] 12. World Bank. Country Classification, 2019. Available from: https://datahelpdesk.worldbank.org/knowledgebase/articles/906519world-bank-country-and-lending-groups Accessed 15 November 2019.

[13] 13. Index Mundi. Countries. Available from: https://www.indexmundi.com/factbook/countries Accessed 15 November 2019.

[14] 14. World Atlas. Countries with Universal Health Care, 2019. Available from: https://www.worldatlas.com/articles/countries-with-universal-health-care.html Accessed 15 November 2019.

[15] 15. Pan American Health Organization. Strategy and Plan of Action on Dementias in Older Persons. 2015. Available from: https://www.paho.org/hq/index.php?option=com_topics&view=rdmore&cid=6286&item=aging-populations&type=mandates&Itemid=40941&lang=en Accessed 15 November 2019.

[16] 16. Dementia warning for the Asia-Pacific region. Lancet Neurol. 2015;14(1):1. 10.1016/S1474-4422(14)70312-6 25496878

[17] 17. Oliva-Moreno J, Trapero-Bertran M, Peña-Longobardo LM, del Pozo-Rubio R. The valuation of informal care in cost-of-illness studies: A systematic review. Pharmaco Econ. 2017;35(3):331-45. 10.1007/s40273-016-0468-y 27848219

[18] 18. Evripidou M, Merkouris A, Charalambous A, Papastavrou E. Implementation of a training program to increase knowledge, improve attitudes and reduce nursing care omissions towards patients with dementia in hospital settings: A mixed-method study protocol. BMJ Open. 2019;9:e030459. 10.1136/bmjopen-2019-030459 PMC6661557 31326938

[19] 19. Lee DW, Seong SJ. Korean national dementia plans: from 1st to 3rd. J Korean Med Assoc. 2018;61(5):298-303.

[20] 20. Nakanishi M, Nakashima T. Features of the Japanese national dementia strategy in comparison with international dementia policies: How should a national dementia policy interact with the public health and social-care systems? Alzheimers Dement. 2014;10(4):468-76. 10.1016/j.jalz.2013.06.005 23954026

